# Overcoming barriers and thresholds – signaling of oligomeric Aβ through the prion protein to Fyn

**DOI:** 10.1186/1750-1326-8-24

**Published:** 2013-07-16

**Authors:** Hansen Wang, Carl He Ren, C Geeth Gunawardana, Gerold Schmitt-Ulms

**Affiliations:** 1 Tanz Centre for Research in Neurodegenerative Diseases, University of Toronto, Tanz Neuroscience Building, 6 Queen’s Park Crescent West, Toronto, Ontario M5S 3H2, Canada; 2 Department of Laboratory Medicine and Pathobiology, University of Toronto, Toronto, Ontario M5S1A8, Canada

**Keywords:** Alzheimer disease, Amyloid β peptide, Fyn, Prion protein, Tau, Excitotoxicity

## Abstract

Evidence has been mounting for an involvement of the prion protein (PrP) in a molecular pathway assumed to play a critical role in the etiology of Alzheimer disease. A currently popular model sees oligomeric amyloid β (oAβ) peptides bind directly to PrP to emanate a signal that causes activation of the cytoplasmic tyrosine kinase Fyn, an essential player in a cascade of events that ultimately leads to NMDA receptor-mediated excitotoxicity and hyper-phosphorylation of tau. The model does not reveal, however, how extracellular binding of oAβ to PrP is communicated across the plasma membrane barrier to affect activation of Fyn. A scenario whereby PrP may adapt a transmembrane topology to affect Fyn activation in the absence of additional partners is currently not supported by evidence. A survey of known candidate PrP interactors leads to a small number of molecules that are known to acquire a transmembrane topology and understood to contribute to Fyn activation. Because multiple signaling pathways converge onto Fyn, a realistic model needs to take into account a reality of Fyn acting as a hub that integrates signals from multiple inhibitory and activating effectors. To clarify the role of PrP in oAβ-dependent excitotoxicity, future studies may need to incorporate experimental designs that can probe the contributions of Fyn modulator pathways and rely on analogous readouts, rather than threshold effects, known to underlie excitotoxic signaling.

## Review

The concomitant accumulation of extracellular aggregates of Aβ peptides and intracellular deposits of the tau protein is a neuropathological hallmark of Alzheimer disease (AD). The details of the molecular biology that connects these AD signature aggregation events are not understood. A currently popular model posits that exposure of cells to oligomeric forms of the amyloid β peptide (oAβ) triggers a cascade of events that causes N-methyl-D-aspartate (NMDA) receptor-mediated excitotoxicity and intracellular deposition of hyperphosphorylated tau (reviewed in [1]).

To date, arguably the most instructive data on this topic may have emerged from the study of selected mouse models. As early as in 1998 it was reported that ablation of Fyn, a non-receptor tyrosine kinase of the larger family of Src family kinases (SFKs), diminishes oAβ toxicity [2]. Around the same time it was shown that SFKs can bind to the N-terminal projection domain of tau [3]. First proposed in 2002, it is now widely accepted that oAβ excitotoxicity also depends on the availability and proper cellular targeting of tau [4,5]. A recent study connected some of the dots by showing that a tau construct engineered to retain the projection domain but lacking the microtubule binding domain misdirected Fyn and prevented oAβ toxicity [6,7].

**Figure 1:**
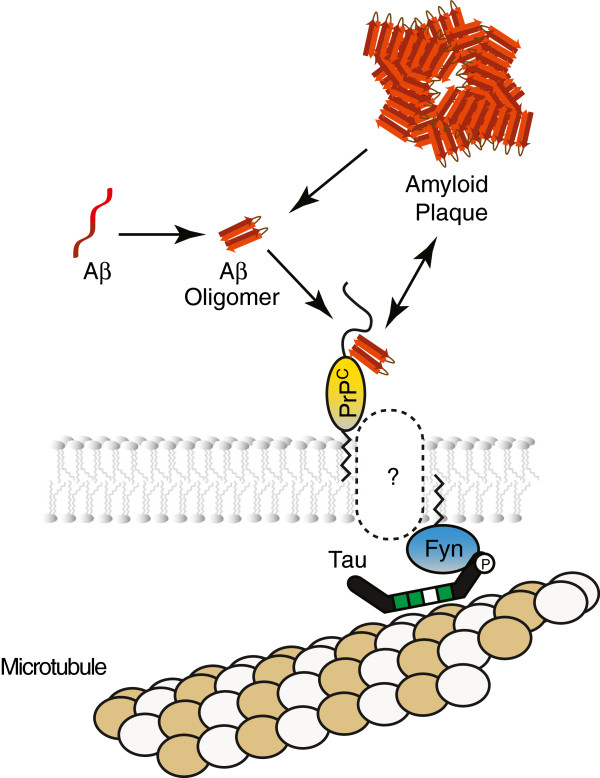
**Schematic depiction of a popular model that ties extracellular oAβ**-**PrP interaction to cytosolic Fyn activation.** The identity of the molecular player that communicates binding of oAβ to PrP into the cell is currently not known. Tau has been ascribed a critical role in the delivery of Fyn to the cytosolic face of the membrane.

Any model of cellular oAβ toxicity needs to explain how extracellular oAβ communicates its presence into the cell. The process can be broken into two main steps: (1) binding of oAβ to the cellular membrane; and (2) signaling across the cellular membrane when oAβ binding has taken place. A large number of theories have been put forward that hypothesize on the nature of the first step and the cellular binding partner involved (see [8,9] for recent reviews). Without a question, amongst the many oAβ candidate receptors proposed, the prion protein (PrP) has of late received the most attention. This may in part be due to the fact that the role of PrP as a candidate oAβ interactor emerged from a hypothesis-free screen, as opposed to conceptually more limited approaches that suggested involvement of many other receptor candidates. At this time, the cumulative binding data available for the oAβ-PrP interaction are the most validated and robust for any oAβ candidate receptor [10-15]. When combined with the aforementioned model a refined scenario emerged according to which oAβ binds to PrP which, in turn, causes activation of Fyn following its delivery to the inner face of the plasma membrane, a step that appears to require tau (Figure 1). However, an ongoing controversy surrounds the significance of oAβ binding to PrP and, more specifically, the question whether this interaction is responsible for downstream neurotoxicity and impairment of long-term potentiation [12,16,17], an electrophysiological surrogate of learning and memory formation. Possible reasons for data discrepancies have been proposed before [1,18,19] and will only be touched in the second half of this article where we highlight aspects that may not have been adequately considered in previous reports.

The focus of this perspective will be on a related question: How is it that PrP, a glycosylphosphatidylinositol (GPI) anchored molecule, tethered to the outer face of the membrane, can activate an intracellular SFK? The need to answer this pressing question has also been noted by others [10], and has led to a situation where several authors of recent papers indicated this knowledge gap in schematic models either with conspicuous question marks or by omitting labels for the corresponding molecular entity altogether (e.g., schematic drawings in [20-22]).

### Overcoming barriers – signaling from PrP to Fyn

A natural place to begin the search for the missing link that ties PrP to Fyn activation is to state the properties a candidate needs to fulfill to serve in this role, namely (i) the ability to bind to PrP; (ii) the existence of a membrane-spanning topology; and (iii) a known role as an activator of SFKs. Although a large number of candidate PrP interactors have been described [23,24], to our knowledge only neural cell adhesion molecules (NCAMs) [25,26], integrin/non-integrin laminin receptors [27], and caveolin-1 [28,29] meet all three of the aforementioned criteria and shall be described in more detail in the following paragraphs. A scenario whereby a PrP-dependent signal may traverse the plasma membrane independent of other transmembrane proteins—if the presence of oAβ induces a well-known but rare transmembrane topology in PrP—will also not be discussed here because, to date, no evidence exists that ties these transmembrane forms of PrP to Fyn activation [19]. Naturally, the decision to limit discussions to PrP interactors with a known Fyn connection is not intended to deny the possible existence of such a connection for other proteins that have been proposed to bind to PrP. The reader will appreciate though that the omission of this operational restriction would have forced a more cursory description of many PrP interactors at the expense of the more focussed perspective that was the aim of this review.

**Figure 2:**
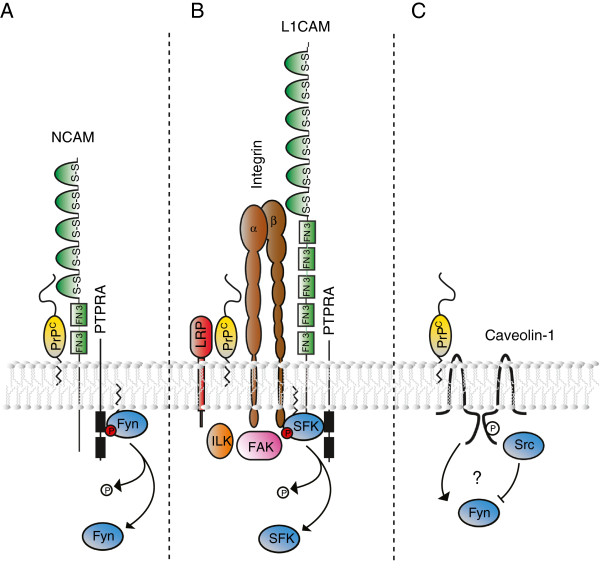
**Possible scenarios and molecular players that may be operative in overcoming the membrane barrier required for PrP**-**to**-**Fyn signaling. (A)** PrP may recruit NCAM into raft domains causing its association with PTPRA, a known activator of Fyn. **(B)** PrP may influence cellular attachment to laminin by interacting with multiple molecules known to play key roles in this cell-to-extracellular matrix interaction. A sub complex of integrins and L1 is known to activate SFKs, again possibly through interaction with PTPRA. **(C)** PrP may modulate activity of Fyn by interacting with Caveolin-1. The consequence of this interaction with regard to Fyn activity status is currently unclear or may not be consistent across experimental paradigms.

NCAMs are members of the immunoglobulin (Ig) superfamily of cell adhesion molecules [30] and are in humans coded on chromosomes 11 (NCAM1) and 21 (NCAM2). Whereas NCAM1 is widely expressed throughout the human brain, NCAM2 is primarily observed in the olfactory bulb. Additional complexity of NCAM expression profiles emerges from alternative splicing and gives rise to both GPI-anchored (p120-NCAM1) and transmembrane forms (p140-NCAM1 and p180-NCAM1) with short cytoplasmic tails. The ectodomain of NCAM1 or NCAM2 comprises five N-terminal Ig-like domains and two membrane-adjacent fibronectin-type 3 (FN3) domains. Given their shared mode of GPI-dependent membrane attachment, it is not surprising to find p120-NCAM1 in proximity of PrP, and even p140-NCAM1 has been shown to be recruited to raft-like domains upon acylation of its cytoplasmic domain [31]. NCAM1 was initially shown to co-immunoprecipitate with PrP following *in vivo* crosslinking of mouse Neuroblastoma cells (Neuro2a) and the direct interaction was shown to depend on a protein-protein interface that mapped to β-strands C and C0 within the two consecutive FN3 modules of NCAM1 and the N-terminus and helix A (residues 144–154) within PrP [26]. Subsequent large-scale interactome studies confirmed not only that NCAM1 is a prominent molecular neighbor of PrP in both cultured neuronal cells and the brain [27,32] but further established that the interaction may recruit NCAM1 into raft-like domains and trigger a signal that leads to activation of Fyn [25], a finding that had partly been foreshadowed by data which indicated that NCAM1 acts in certain experimental paradigms upstream of Fyn [33]. A hint at the mechanism by which NCAM1–lacking an SFK activation domain of its own–may activate Fyn then emerged from studies that documented a dependency of NCAM1-mediated neuritic outgrowth on receptor-type tyrosine-protein phosphatase α (PTPRA), also known as RPTPα [34]. The finding complemented prior data which had documented that PTPRA can activate SFKs by removing their inhibitory C-terminal tyrosine phosphorylation [35,36] and was operative in transmembrane Fyn activation emanating from contactins [35] or integrins [37]. PTPRA may itself get activated in this context by phosphorylation at two serine acceptor sites (S180/204) [38,39]. One scenario suggests PTPRA is phosphorylated by protein kinase C in this manner once this kinase has been recruited through PrP^C^-dependent clustering of NCAM1 [40] (Figure 2A).

Non-integrin- and integrin-based laminin interactions: PrP^C^ has repeatedly been shown to interact with ribosomal protein SA (RPSA), also known as the 67 kDa laminin receptor [41], a cellular non-integrin receptor of laminin. The link to the extracellular matrix protein laminin was further strengthened when a quantitative interactome analysis revealed PrP to co-purify with VLA-6 [27], a heterodimeric α6β1 integrin complex also known to mediate cellular attachment to laminin (Figure 2B). Integrins represent a large family of transmembrane receptors with well-established roles in mediating cellular contacts with extracellular matrices. Mammalian genomes code for 18 α and 8 β subunits of integrins that assemble into more than 24 known functional α/β heterodimers with specialized roles in extracellular matrix recognition [42]. In addition to the α6β1 complex, PrP was shown to also co-enrich with αV, an integrin subunit with a role in vitronectin recognition [27]. Although no direct connection of RPSA to SFK has been established, in addition to having been independently identified as laminin and PrP interactors, RPSA and integrins are known to interact with each other [43-45] and integrins are well-known to signal through SFKs. The exact mechanisms by which integrin heterodimers, lacking conspicuous intracellular signaling domains, activate SFKs are less well understood. It is, however, known that extracellular matrix interactions of integrin complexes trigger conformational rearrangements within the integrin heterodimers that expose cytosolic epitopes which, in turn, may sequester signaling factors. A more indirect mechanism of α6β1 integrin signaling may rely on its association with L1. Similar to NCAM, L1 is a cell adhesion molecule of the immunoglobulin superfamily that was shown to co-enrich with PrP [27]. The existence of macromolecular complexes comprising integrins and L1 is no controversial issue [46] and has been shown to rely on the affinity of integrins toward an RGD-amino acid motif within the Ig-like domain 6 in L1 [47]. However, the precise mechanism by which the complex activates Fyn or other SFKs is still somewhat unclear. Some authors have suggested activation may be based on the aforementioned tyrosine phosphatase PTPRA [48,49] (as in Figure 2A).

Caveolins are small membrane-embedded proteins that assemble into clathrin-like lattices and give shape to so-called caveolae, 50–100 nanometer diameter membrane domains enriched in cholesterol and sphingolipids [50]. Caveolae play a role in diverse biological activities, including receptor-independent endocytosis, transcytosis and the docking of viruses and toxins, and also serve as integration hubs for cell signaling [50]. Of the three known caveolins (caveolin-1, -2 and −3) the caveolin-1 gene product has been implicated in PrP^C^-dependent signaling to Fyn. Early work indicated that a cytosolic membrane-proximal region of caveolin-1 (residues 82–100) can bind Src or Fyn, leading to suppression of tyrosine kinase activity [51]. Subsequent reports described that antibody-mediated crosslinking of PrP^C^ in a cell model of neuronal differentiation (1C11) [28] or in a hypothalamic neuronal cell line (GN11) [52] can lead to caveolin-1 dependent Fyn activation. A more recent study conducted with the PC12 cell model suggested yet another twist to the role of caveolin-1 in PrP^C^-dependent Fyn signaling. The authors proposed PrP-mediated crosslinking may rely on integrins for signal transmission across the membrane but that a phosphoepitope generated on caveolin-1 serves in this process as a docking site to recruit Src kinase and inactivate Fyn [53] (Figure 2C)*.*

Needless to say, the short list of molecules discussed in the preceding paragraphs may not yet contain the missing link responsible for oAβ-mediated signaling from PrP to Fyn. Therefore, efforts to validate the role of these candidates need to be pursued in parallel to unbiased searches for novel interactors of PrP in its oAβ-bound state based on hypothesis-free discovery platforms (e.g., affinity capture followed by mass spectrometry or mammalian two-hybrid system derivatives). Conclusive evidence that a given PrP interactor represents the missing link will not come easy as the field is lacking robust experimental paradigms. Even some of the most widely used experimental setups that recapitulate oAβ-dependent tau hyperphosphorylation, i.e., the treatment of rodent primary neurons or hippocampal slice cultures with oAβ, are far from trivial and relatively refractory to manipulations. A first meaningful step, however, would be to show that the knockdown of a missing link candidate protein in these paradigms reduces oAβ-dependent Fyn activation. These studies should be complemented with efforts to map the precise protein-protein interfaces that mediate binding of a candidate missing link to PrP and Fyn. Once this hurdle has been taken, candidate genes will need to be validated in rodent AD models, an objective made easier with recent advances in gene targeting strategies [54]. Aside from the challenge to identify a suitable AD model, these experiments could, however, be confounded by developmental or compensatory systems biology. To be able to correlate the knockout of a candidate gene with a reduction in oAβ-dependent Fyn activation experimental approaches need to be either sensitive (if analyses are undertaken on brain tissue homogenates) or, preferably, offer single-cell resolution, because the molecular phenotype is likely to manifest in only a subset of cells.

### Overcoming thresholds – molecular networks, signaling hubs and cellular programs

So far in this article protein-protein interactions that PrP^C^ engages in have been described as if they existed in isolation or followed a strictly linear and sequential signaling logic. Although useful at a certain level, this approach does not do justice to a reality of a highly intricate cell biology governed by molecular networks, convergence and branching of signaling pathways and highly coordinated cellular programs than can choreograph the molecular biology of a large number of proteins concomitantly [55,56]. In the second part of this review an attempt is made to capture some of this complexity.

Although many proteins that PrP^C^ is surrounded by in the brain have been identified by large-scale PrP^C^-centric interactome studies in mice [32,57], little is known about the authentic molecular neighborhood of this protein in a given brain cell relevant for AD. In the absence of this information, data from a quantitative PrP^C^ interactome analysis in mouse neuroblastoma cells may offer the next best glimpse into the molecular environment of PrP^C^ in a specific cell type [58]. According to this study PrP^C^ resides sufficiently close to a few dozen different proteins to facilitate its covalent intermolecular crosslinking to them. As expected for a protein that is inserted into the plasma membrane by a glycosylphosphatidylinositol anchor, the PrP interactome is dominated by ER- and Golgi-resident proteins, as well as other membrane proteins. Of note, a body of literature suggests that a substantial proportion of PrP^C^ resides within cells in membrane (lipid) rafts [59,60], detergent-resistant membrane domains rich in cholesterol and sphingolipids, and it has been proposed that this localization of PrP^C^ is critical for its ability to mediate oAβ toxicity [61]. Although the organizing principles that drive the composition of membrane rafts are still a matter of debate [62], it is likely that the molecular neighborhood of PrP^C^ within these raft domains is not only governed by PrP^C^-lipid interactions but also by the relative affinity of PrP^C^ towards other raft-resident proteins [63]. On the basis of these considerations it may not surprise that a majority of the aforementioned PrP^C^ interactors (e.g., NCAM [31], the laminin recepetor precursor [64], integrins [65] and caveolin-1 [28]) have also been localized—at least transiently—in membrane rafts or in the aforementioned caveolae, a specialized subset of membrane rafts. Consequently, signals emanating from PrP^C^ and leading to Fyn activation are unlikely to solely rely on the interaction of PrP^C^ with just one type of binding partner but, instead, when circumstances are permissive (see below), may involve, to varying degrees, and in parallel, multiple of the signaling scenarios outlined above (Figure 2).

A first indication that concerted action of multiple PrP molecules might be needed for Fyn activation provided the aforementioned antibody-mediated PrP crosslinking data in the 1C11 neuronal differentiation cell model [28]. A more general role of receptor clustering in Fyn activation came to the fore when it was subsequently shown that several other receptors could similarly only activate Fyn when clustered and associated with membrane rafts [40,66,67]. Whereas there is general agreement that only oAβ, not mAβ, binds to PrP [12,13,29,68], less clear is whether oligomeric forms of Aβ illicit neurotoxicity chiefly on account of their multi-valency and, thus, ability to promote Fyn activation through clustering of PrP.

**Figure 3:**
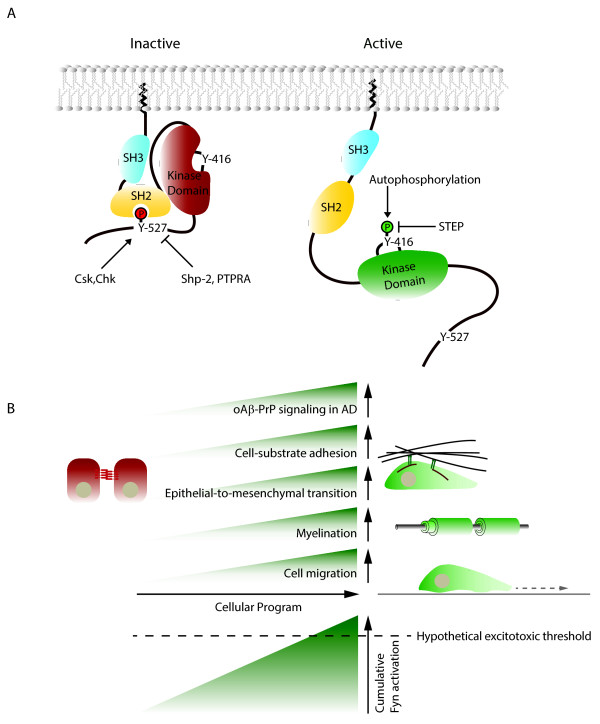
**Fyn’s role as a signal integration hub involved in a number of cellular programs need to be taken into account when assessing role of PrP in oAβ excitotoxicity paradigms. (A)** Fyn belongs to the family of non-receptor-type protein tyrosine kinases referred to as Src family kinases (SFKs), of which five members (Src, Fyn, Yes, Lck and Lyn) are known to be expressed in the human brain, and Fyn and Src are of primary interest in the context reviewed here [76]. All SFKs share a modular domain organization composed of a Src homology 2 (SH2) domain sandwiched between an N-terminal Src homology 3 (SH3) domain and a C-terminal tyrosine kinase domain. The inactive state of these kinases can be stabilized by an intramolecular interaction that forms when the SH2 domain binds to a C-terminal tyrosine-phosphate (Y527). Full activation requires dephosphorylation of this inhibitory phosphate and autophosphorylation within the activation loop in the tyrosine kinase domain. Various tyrosine kinases and phosphatases have been shown to control occupancy of these critical acceptor sites. Thus, SFK activity is negatively regulated through Y527 phosphorylation by C-terminal Src Kinase (CSK) or CSK homologous kinase (CHK) and through dephosphorylation of Y416 by striatal-enriched tyrosine phosphatase (STEP) [77]. Positive effectors of SFK activity are the receptor-type protein tyrosine phosphatases Shp-2 [78,79] and PTPRA [35,36] that were shown to selectively remove the inhibitory Y527 phosphate. **(B)** Cellular programs known to modulate Fyn activation levels. Upon oAβ binding to PrP^C^ and signaling to Fyn, the cumulative Fyn activation level will be reflective of the cell type, its developmental status and the programs executed in the cell.

Because Fyn itself acts as a molecular hub on which signals from multiple pathways converge, a realistic evaluation of its activation state needs to consider additional inputs known to modulate Fyn activity. Thus, the signaling outcome of oAβ binding to PrP^C^ will also be impacted by the status of STEP2, Shp2 and a few other kinases/phosphatases that, taken together, influence the activity level of Fyn (see Figure 3). When viewed in this light, a number of seemingly disconnected observations may begin to make sense. It has, for example, been reported that antibodies specific for the β1 subunit of integrins can block oAβ-mediated toxicity in primary neuronal cultures [69]. In light of the signal hub role of Fyn and an absence of evidence that oAβ can bind to integrins, this observation could reflect an influence of integrin biology on Fyn-related signaling (see Figure 2B). Similarly, inhibitors of amylin receptors have repeatedly been shown to prevent oAβ-dependent toxicity but there is no compelling data that oAβ can bind to amylin receptors [70,71]. Amylin receptors, however, are known to initiate a signaling cascade that utilizes the second messenger cAMP to activate protein kinase A (PKA) [72-74], which in turn would be predicted to alter the activity of STEP2 and, thereby, modulate the activation status of Fyn [75].

A similar integration of inhibitory and activating signals likely occurs at multiple points of convergence of pathways involved in NMDA receptor-mediated excitotoxicity, including an increasingly understood but highly intricate biology that underlies the modulation of expression levels, subcellular positioning and posttranslational activation of NMDA receptors themselves [75,80]. For example, the ability of PKA to modulate NMDA receptor activation may not only operate by impacting Fyn activity through regulation of STEP2 but may, in addition, influence Fyn-dependent activation of NMDA channels by controlling its association with Rack1. To this end, it has been demonstrated that PKA phosphorylation of Rack1 causes its translocation, thereby freeing cytosolic phospho-acceptor sites within the channel’s NR2B subunit from a steric hindrance to Fyn phosphorylation (reviewed in [75]). Thus, the restricted focus on Fyn signal integration in this review does not reflect a view that Fyn is the only, or even the most important, signal integration hub on pathway to excitotoxic signaling in AD.

Given that Fyn is considered to exist in cells predominantly in an inactive state, the question arises as to which broader cellular programs cause its activation. The kinase has been linked to diverse biological functions. Whereas early work on Fyn emphasized possible neurological and immunological functions, interest has shifted toward other roles that include its involvement in neurite elongation in oligodendrocytes [81] and neurons [34], myelination [82,83], and the cellular reprogramming that can lead to epithelial-to-mesenchymal transition [84,85] or cancer [86,87]. Interestingly, the molecular biology underlying a subset of these cellular programs is surprisingly similar. Commonalities are a reliance on signaling through membrane lipid raft domains, morphogenetic rearrangements that depend on cell-to-substrate adhesion and even the shared involvement of a strikingly overlapping set of molecular players. It is, for example, increasingly being understood that the cellular program which drives myelination depends on an intricate molecular network that includes GPI-anchored molecules, a subset of integrins, L1 and Fyn [82,83,88,89]. Similarly, the transient interactions that a motile cell forms toward its substrate or that a growing neurite engages in as it is probing its environment have been known for some time to make use of signaling through rafts and strongly depend on Fyn activation [25,33,81]. Finally, it is emerging that many of the same proteins are also involved in the execution of epithelial-to-mesenchymal transitions, cellular programs relevant for both normal development during ontogenesis and abnormal motility of cells seen during invasive stages of cancers [84,86,87]. It is not known at this time if the molecular overlaps of these cellular programs also offer a starting point for understanding why PrP deficiency leads to a myelin maintenance defect affecting peripheral nerves in mice [90] but causes a defect in epithelial-to-mesenchymal transition during the early developmental gastrula stage in zebrafish [91].

Returning to the main theme of this article, the question arises if a cell that is executing one of the cellular programs which involve Fyn activation is more susceptible to oAβ toxicity. Similarly, can testable hypotheses be derived from the aforementioned considerations that may bridge the seemingly irreconcilable positions of the two camps of scientists who either insist PrP to be critical or not necessary at all for oAβ-mediated neurotoxicity? It is noteworthy that the apparent controversy surrounding the involvement of PrP^C^ in mediating oAβ toxicity is based on models that measure threshold effects, i.e., the excitotoxic activation of NMDA receptors in similar but non-identical experimental paradigms. In such a scenario, small differences in Fyn activation levels can manifest as dramatic differences in outcome. A productive path forward to reconcile the apparent contradictions in observations, therefore, might be to base future analyses on read-outs that are by their nature analogous and quantifiable, e.g., the ratio of levels of activated Fyn to total Fyn. A first step in this direction might be to establish the relative contributions converging pathways exert on the cumulative overall Fyn activation levels. In other words, does the oAβ effect on PrP represent a dominant or minor Fyn activation pathway? Future attempts to push back oAβ-mediated neurotoxicity may be futile if therapeutic interventions concentrate on only one of multiple pathways that converge on Fyn. Unless the overall level of Fyn activation is reduced, its excitotoxic threshold may be overcome through cellular changes that manifest with age or altered metabolism, or an acute imbalance of a given Fyn-activation pathway based on inflammation or injury.

## Conclusions

The ongoing debate surrounding the proposed involvement of PrP in a central signaling pathway thought to connect oAβ exposure of cells to Fyn activation has repolarized the AD research field. Insights into the molecular mechanism by which PrP may communicate oAβ binding into the cell are urgently needed. This review distilled from a broad literature on PrP protein-protein interactions NCAM, integrins and, possibly, caveolin-1 as molecules worth investigating for a possible involvement in this step. Validation efforts for these proteins could be pursued in parallel to research aimed at revealing additional candidates. A revised model should also consider the role clustering of PrP may play in oAβ-mediated Fyn activation. Finally, a reality of Fyn operating as a signal integration hub demands experimental designs that can more fully capture the level of Fyn activation and suggests attention should also be paid to alternative pathways and cellular programs Fyn participates in.

## Abbreviations

AD: Alzheimer disease; GPI: glycosylphosphatidylinositol; NCAM: neural cell adhesion molecule; NMDA: N-methyl-D-aspartate; oAβ: oligomeric amyloid β; PrPC: cellular prion protein; PTPRA: receptor-type tyrosine-protein phosphatase α; RPSA: ribosomal protein SA; SFK: Src family kinase.

## Competing interests

All authors declare no competing interests.

## Authors’ contributions

GS conceptualized and drafted a first version of the manuscript. All authors contributed to the interpretation of data, the design of illustrations and the revising of the manuscript. All authors read and approved the final manuscript.

## Authors’ information

Dr. Hansen Wang is a Senior Postdoctoral Researcher at the University of Toronto. He is currently investigating signaling downstream of oAβ. Carl He Ren is a Graduate student at the University of Toronto. He is studying the physiological role of the prion protein. Dr. Geeth Gunawardana is a Postdoctoral Research Fellow at the University of Toronto. His research focuses on the role of the tau protein in oAβ-dependent excitotoxic signaling. Dr. Gerold Schmitt-Ulms trained in the laboratory of Dr. Stanley Prusiner at the University of California, San Francisco, before joining the Tanz Centre for Research in Neurodegenerative Diseases (Tanz CRND), University of Toronto, in 2003. He is an Associate Professor and Graduate Faculty of the Department of Laboratory Medicine & Pathology (LM&P). Dr. Schmitt-Ulms’ work contributes to two strands of research at the interface of proteomics and neurodegenerative disease research: the development of strategies for the study of protein interactions and the application of these strategies to dissect the early etiology of Alzheimer disease and prion diseases.
